# Identifying Cancer-Related lncRNAs Based on a Convolutional Neural Network

**DOI:** 10.3389/fcell.2020.00637

**Published:** 2020-08-11

**Authors:** Zihao Liu, Ying Zhang, Xudong Han, Chenxi Li, Xuhui Yang, Jie Gao, Ganfeng Xie, Nan Du

**Affiliations:** ^1^Department of Oncology, Medical School of Chinese PLA, Chinese PLA General Hospital, Beijing, China; ^2^Department of Oncology, The Fourth Medical Center, Chinese PLA General Hospital, Beijing, China; ^3^Department of Pharmacy, Heilongjiang Province Land Reclamation Headquarters General Hospital, Harbin, China; ^4^College of Bioinformatics Science and Technology, Harbin Medical University, Harbin, China; ^5^Department of Oncology, Southwest Hospital, Army Medical University, Chongqing, China

**Keywords:** long non-coding RNA (lncRNA), cancer, convolutional neural network (CNN), deep belief network (DBN), machine learning

## Abstract

Millions of people are suffering from cancers, but accurate early diagnosis and effective treatment are still tough for all doctors. In recent years, long non-coding RNAs (lncRNAs) have been proven to play an important role in diseases, especially cancers. These lncRNAs execute their functions by regulating gene expression. Therefore, identifying lncRNAs which are related to cancers could help researchers gain a deeper understanding of cancer mechanisms and help them find treatment options. A large number of relationships between lncRNAs and cancers have been verified by biological experiments, which give us a chance to use computational methods to identify cancer-related lncRNAs. In this paper, we applied the convolutional neural network (CNN) to identify cancer-related lncRNAs by lncRNA's target genes and their tissue expression specificity. Since lncRNA regulates target gene expression and it has been reported to have tissue expression specificity, their target genes and expression in different tissues were used as features of lncRNAs. Then, the deep belief network (DBN) was used to unsupervised encode features of lncRNAs. Finally, CNN was used to predict cancer-related lncRNAs based on known relationships between lncRNAs and cancers. For each type of cancer, we built a CNN model to predict its related lncRNAs. We identified more related lncRNAs for 41 kinds of cancers. Ten-cross validation has been used to prove the performance of our method. The results showed that our method is better than several previous methods with area under the curve (AUC) 0.81 and area under the precision–recall curve (AUPR) 0.79. To verify the accuracy of our results, case studies have been done.

## Introduction

Four to nine percent of the sequences' transcription are long non-coding RNAs (lncRNAs) in mammalian genomes (Canzio et al., 2019; Ji et al., 2019). lncRNA was regarded as the noise of genome transcription and did not have biological functions at first. However, an increasing number of studies have reported that lncRNA is widely (Robinson et al., 2019) involved in chromosome silencing, genomic imprinting, chromatin modification, transcriptional activation, transcriptional interference, and nuclear transport (Cheng et al., 2018a). Recently, it has been proven to be associated with many kinds of cancers.

The secondary structure, spliced form, and subcellular localization of most lncRNAs are conserved (Karner et al., 2020), which is very important for lncRNA to execute functions. However, compared to the functions of microRNAs (miRNAs) and proteins, the function of lncRNA is more difficult to determine. According to the position of lncRNA in the genome relative to protein-coding genes, it can be divided into five types: sense, antisense, bidirectional, intronic, and intergenic.

Many researchers have found lncRNAs play an important role in cancers (Avgeris et al., 2018; Cheng et al., 2018b; Zhao et al., 2020) and neurodegenerative diseases (Peng and Zhao, 2020) as other biological molecules (Zhang T. et al., 2017; Bai et al., 2019; Cheng et al., 2019a; Liang et al., 2019). Although many researchers have verified many associations between lncRNAs and cancers by biological experiments, compared with our knowledge about disease-related genes, we still do not know enough about disease-related lncRNAs. Considering the time and money cost of finding disease-related lncRNAs, more and more researchers tend to use computational methods to identify disease-related lncRNAs. These methods could be divided into three categories: machine learning methods, network methods, and other methods.

Machine learning methods build models based on the similarities of diseases or lncRNAs and their biological characteristics (Cheng, 2019; Cheng et al., 2019b; Zeng et al., 2019; Zou et al., 2019). Lan et al. (2017) developed the lncRNA–disease association prediction (LDAP) which is a method based on bagging support vector machine (SVM) to identify lncRNA–disease associations. They used similarities of lncRNAs and diseases as the features. Yu et al. (2019) developed collaborative filtering naive Bayesian classifier (CFNBC) based on naive Bayesian. They integrated miRNA–lncRNA associations, miRNA–disease associations, and lncRNA–disease associations to infer more lncRNA–disease associations. Considering the discriminative contributions of the similarity, association, and interaction relationships among lncRNAs, disease, and miRNAs, Xuan et al. (2019a) developed a dual convolutional neural network (CNN) with attention mechanisms to predict disease-related lncRNAs.

Network methods are the most common way to identify associations between diseases and lncRNAs nowadays (Gu et al., 2017; Yu et al., 2017; Zhang J. et al., 2017; Kuang et al., 2019; Wang L. et al., 2019; Liu et al., 2020). This kind of method would build one or multiple networks to infer new information. Wang L. et al. (2019) built a lncRNA–miRNA–disease interactive network and used their novel method “LDLMD” to predict associations between lncRNAs and diseases. Sumathipala et al. (2019) used a multilevel network topology which includes lncRNA–protein, protein–protein interaction, protein–disease relationship to use network diffusion algorithm to predict disease-related lncRNAs. The graph convolutional network (GCN) and CNN were used on a lncRNA–miRNA–disease network by Xuan et al. (2019b). Deng et al. (2019) built lncRNA similarity network, disease similarity network, miRNA similarity network, and their associations. Then, they calculated the meta-path and feature vector for each lncRNA–disease pair in the heterogeneous information network.

Other methods may borrow the feature extraction method or similarity conjecture of network methods, but the core of this method is matrix decomposition or matrix completion. Lu et al. (2019) developed the geometric matrix completion lncRNA–disease association (GMCLDA) which is a method based on geometric matrix completion. They calculated disease similarity based on Disease Ontology (DO) and calculated the Gaussian interaction profile kernel similarity for lncRNAs. Then they inferred disease-related lncRNAs based on the association patterns among functionally similar lncRNAs and similar diseases. Wang Y. et al. (2019) proposed a weighted matrix factorization to capture the inter(intra)-associations between different types of nodes. Then, they approximated the lncRNA–disease association matrix using the optimized matrices and weights to predict disease-related lncRNAs. Locality-constrained linear coding label propagation Latent Dirichlet Allocation (LLCLPLDA) was developed by Xie et al. (2019). Firstly, local-constraint features of lncRNAs and diseases were extracted by locality-constrained linear coding (LLC). Then, they predicted disease-related lncRNAs by label propagation (LP) strategy.

However, previous methods did not consider the regulating target gene expression of lncRNA, which is an important function of lncRNA and plays an important role in associations between lncRNAs and diseases. In addition, deep learning methods are an important tool and have shown their power in bioinformatics (Chen et al., 2019; Lv et al., 2019; Wei et al., 2019; Wu et al., 2019; Zhao et al., 2019a,b,c). Therefore, in this paper, we used this information as features of lncRNA. In addition, the expression of lncRNA in different tissues were also used as the features of lncRNA. Then, the deep belief network (DBN) was used to encode, and the CNN was used to classify.

## Methods

### Feature Extraction

#### Tissue Expression Specificity of Long Non-coding RNA

Compared with protein-coding genes, lncRNA shows strong tissue specificity. The specificity of lncRNAs in different kinds of tissues and cell types has been proven by many biological experiments. The different expression also plays an important role in essential cellular processes. Sasaki et al. (2007) tested the expression of lncRNAs in 11 different tissues and found 67% lncRNAs exhibited tissue-specific expression and 29% of lncRNAs were only expressed in one discrete tissue. Therefore, the expression of lncRNAs in different tissues were used as the features.

We obtained the expression of lncRNAs in 13 different tissues which included adipose, adrenal, breast, colon, heart, kidney, liver, lung, lymph node, ovary placenta, prostate, testis, and thyroid.

Therefore, the dimension of each lncRNA's expression feature is 1 * 13.

#### Target Gene of Long Non-coding RNA

Quantitative reverse transcriptase-polymerase chain reaction (qRT-PCR) and Western blot were used to test the different expression genes after knocking down or overexpressing lncRNAs.

We obtained target genes of lncRNA from LncRNA2Target (Jiang et al., 2015).

**Figure 1:**
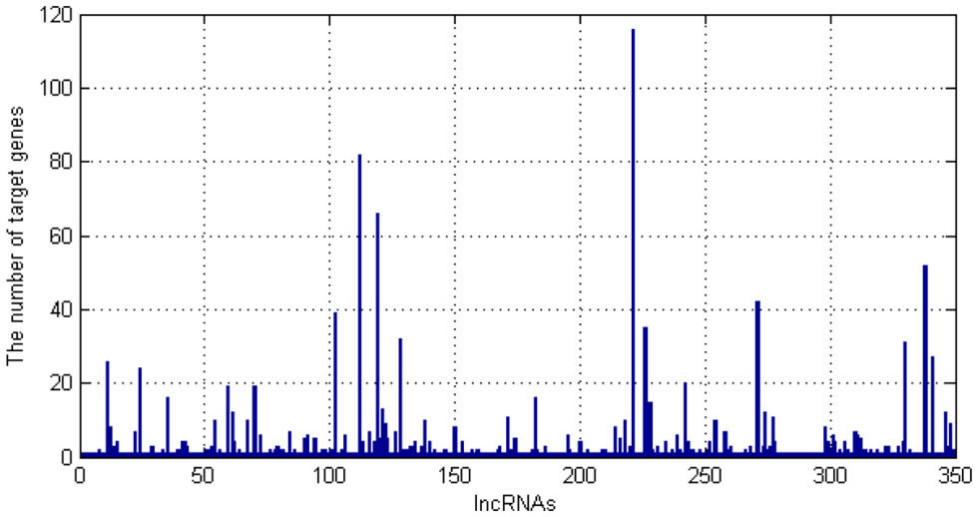
The number of target genes for each long non-coding RNA (lncRNA).

As we can see in Figure 1, there are 349 kinds of lncRNAs. One lncRNA has more than 100 target genes. Then, we draw the distribution of the number of target genes corresponding to lncRNA.

**Figure 2:**
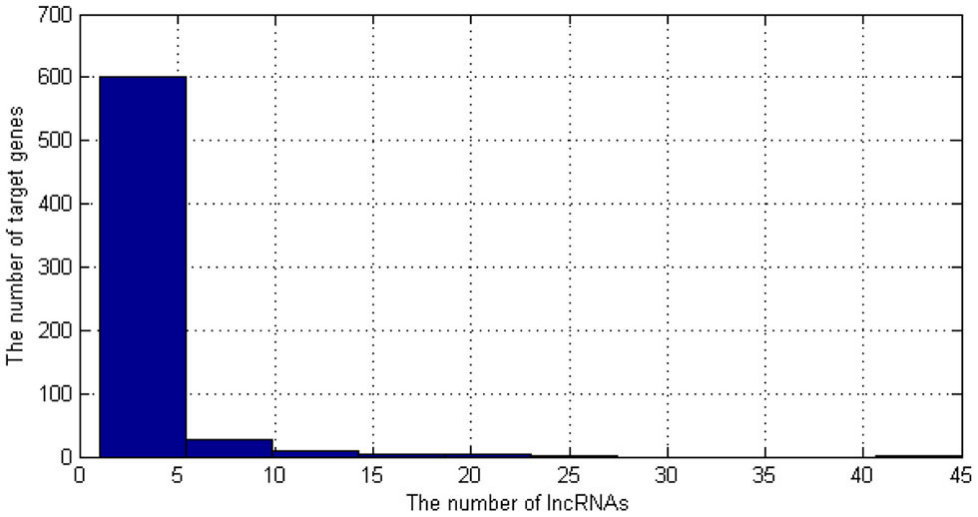
The distribution of the number of target genes. lncRNA, long non-coding RNA.

As shown in Figure 2, most of the target genes are corresponding to less than five lncRNAs. Therefore, if we used them to be the features of lncRNAs, the features would be sparse. Therefore, we only select the most common target genes to be the features. The genes which are corresponding to more than five lncRNAs were selected as the features of lncRNAs. There are 45 kinds of genes. Then, we need to encode these genes.

(1) $$\begin{array}{l} F=\left[G_{1},G_{2},\cdots ,\;G_{45}\right] \end{array}$$

where *G*_1_ denotes the first gene of these 45 genes, and F denotes the feature of lncRNA. For each lncRNA, if *G*_1_ is the target gene of it, then *G*_1_ = 1, otherwise *G*_1_ = 0.

Therefore, the dimension of each lncRNA's target gene feature is 1 * 45.

### Deep Belief Network

The DBN can effectively learn complex dependencies between variables (Zhao et al., 2019d). The DBN contains many layers of hidden variables, which can effectively learn the internal feature representation of the data and can also be used as an effective non-linear dimensionality reduction method.

When the observable variables are known, the joint posterior probabilities of the hidden variables are no longer independent of each other, so it is difficult to accurately estimate the posterior probabilities of all hidden variables. The posterior probability of early DBN is generally approximated by Monte Carlo method, but its efficiency is relatively low, which makes its parameter learning difficult. In order to effectively train the DBN, we convert the sigmoid belief network of each layer to a restricted Boltzmann machine (RBM). The advantage of this is that the posterior probabilities of the hidden variables are independent of each other, which makes it easy to sample. In this way, the DBN can be regarded as being stacked from top to bottom by multiple RBMs, and the hidden layer of the Lth RBM is used as the observable layer of the L + 1th RBM. Further, the DBN can be trained quickly by layer-by-layer training, that is, starting from the bottom layer and training only one layer at a time until the last layer. The specific layer-by-layer training process is to train the RBM of each layer in turn from bottom to top. Assuming we have trained the RBM in the first L-1 layer, we can calculate the conditional probability of the bottom-up hidden variables:

(2) $$\begin{array}{l} p\left(h^{\left(i\right)}|h^{\left(i-1\right)}\right)=\sigma \left(b^{\left(i\right)}+W^{\left(i\right)}h^{\left(i-1\right)}\right) \end{array}$$

where *b*^(*i*)^ is the bias of ith layer of RBM. *W*^(*i*)^ is the connection weight. *h*^(*i*)^ is the ith layer of RBM.

The process of training DBN is as follows:

**Table:** 

Input : train dataset ${\hat{v}}^{\left(n\right)}$, learning rate λ
Output: weight matrix *W*^(*l*)^, bias *a*^(*l*)^ and *b*^(*l*)^
For l = 1:L
Initialization: *W*^(*i*)^, *a*^(*l*)^, *b*^(*i*)^ = *0*
Sample from train dataset *ĥ*^(0)^
For i = 1: *l−1*
Sample *h*^(*i*)^ based on *p*(*h*^(*i*)^|*ĥ*^(*i*−1)^)
End
Set *h*^(*i-1*)^as the train sample to train lth layer of
RBM
End

Since the dimension of expression feature and target gene feature are different, we should reduce the dimension of target gene feature and make it the same as the expression feature's. Therefore, in this paper, two layers of RBM were used to build a DBN model.

The number of nodes of the two layers was 32 and 12, respectively. Sigmoid function was used as the activation function.

(3) $$\begin{array}{l} \sigma \left(x\right)=\frac{1}{1+e^{-x}} \end{array}$$

Therefore, the dimension of final features is 2 * 13.

(4) $$\begin{array}{l} F=\left[\begin{array}{c} G_{1},G_{2},\cdots ,\;G_{\text{13}}\\ E_{1},E_{2},\cdots ,\;E_{\text{13}} \end{array}\right] \end{array}$$

where *G*_1_, *G*_2_, ⋯, *G*_13_ denotes target gene feature after DBN, and *E*_1_, *E*_2_, ⋯, *E*_13_ denotes the expression of lncRNAs in 13 different tissues.

### Convolutional Neural Network

The power of CNN in dealing with bioinformatic problems has been proven by many researchers. We selected CNN as the classifier based on two reasons. (1) The dimension of features is 2 * 13, which can be regarded as an image. (2) The outstanding performance of CNN in image classification.

There are five layers in our CNN model. The structure of CNN is shown as Table 1.

**Table 1:** The structure of convolutional neural network (CNN).

**Layers**	**Parameter**
Convolutional layer	Filter = 64 kernel size = (1,4) Activation function = tanh
Pooling layer	pool size = (2,2) Activation function = tanh
Convolutional layer	Filter = 128 kernel size = (1,2) Activation function = tanh
Pooling layer	pool size = (1,2) Activation function = tanh
Fully connected layer	Units = 512 Activation function = tanh
Output	Units = 2 Activation function = sigmoid

### Work Frame

**Figure 3:**
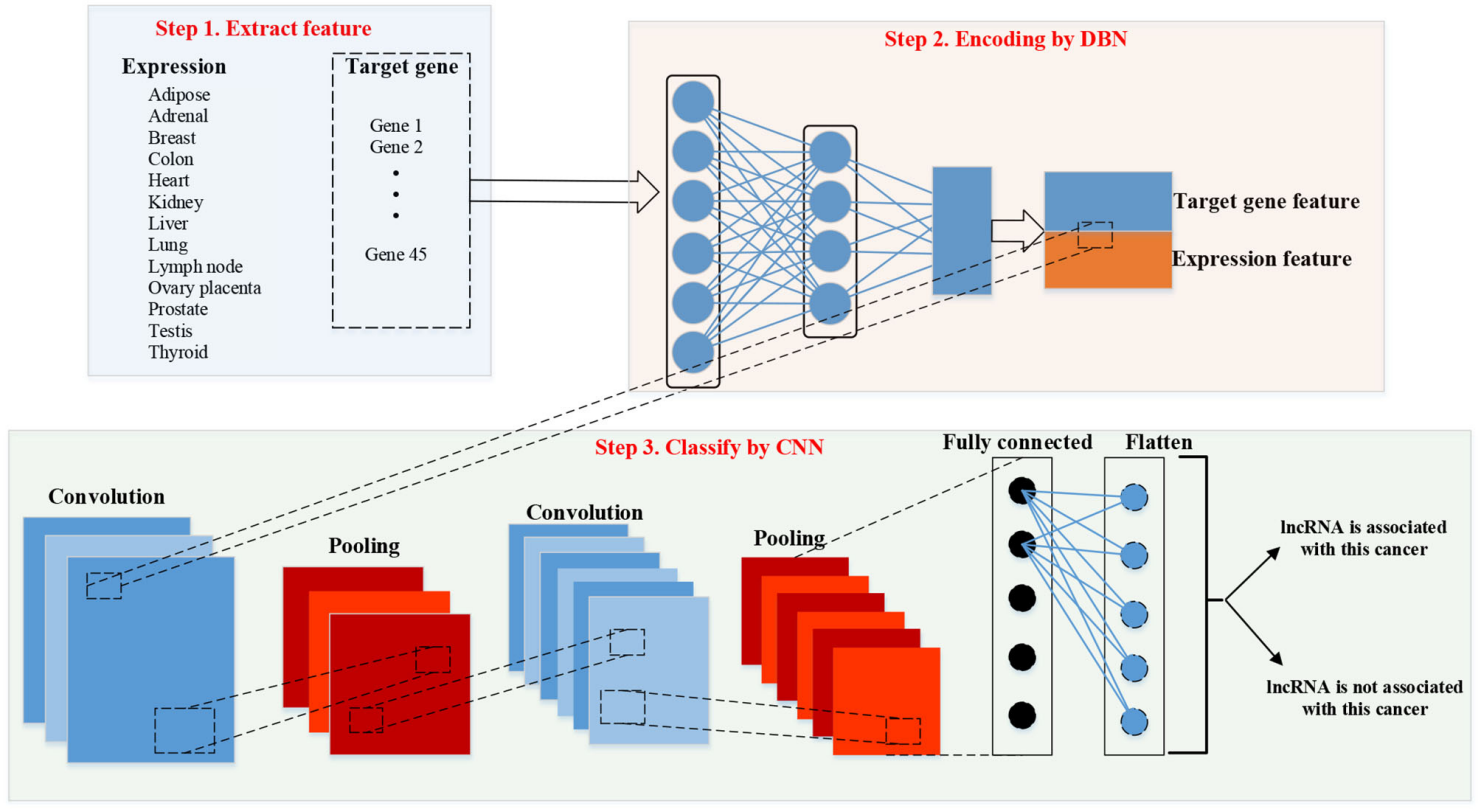
Work frame of deep belief network (DBN)–convolutional neural network (CNN). lncRNA, long non-coding RNA.

Figure 3 shows the work frame of our method “DBN–CNN.” There are three steps of our methods. Firstly, we should extract features of lncRNAs. There are two parts of features: expression feature and target gene feature. Then, DBN was used to encode the target gene feature. After encoding, the two kinds of features were combined together. Finally, CNN was used to classify.

## Results

### Data Description

**Figure 4:**
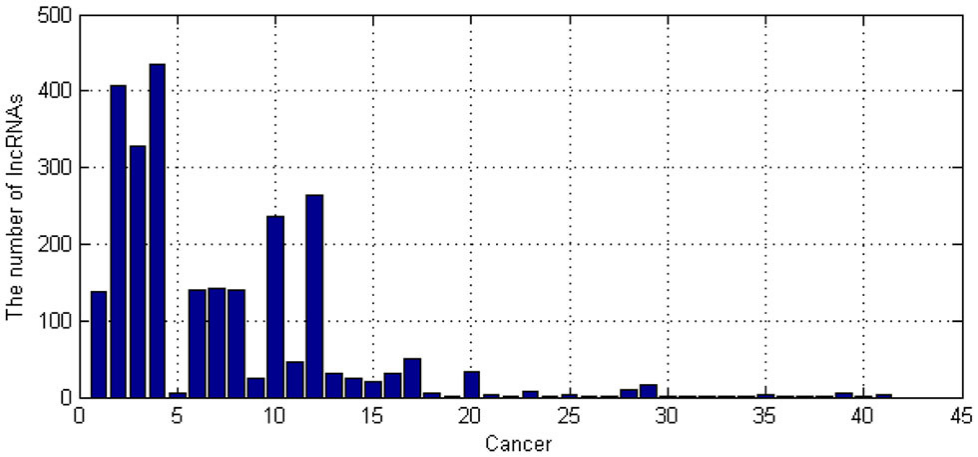
The number of long non-coding RNAs (lncRNAs) for each cancer.

The known associations between lncRNA and diseases were obtained from LncRNADisease database (Bao et al., 2019). We totally obtained 41 kinds of cancer-related lncRNAs. The number of their corresponding lncRNAs is shown as Figure 4.

As shown in Figure 4, People's understanding of cancer-related lncRNAs varies widely. We have known more than 100 lncRNAs for some cancers, but few lncRNAs are known for some cancers. To better build our model, we only selected cancers which have more than 20 related lncRNAs. Therefore, 16 kinds of cancers were selected.

The target genes of lncRNAs were obtained from LncRNA2Target database. We have discussed about this in section *Target Gene of Long Non-coding RNA*.

The expression of lncRNAs in 13 different tissues was obtained from NON-CODEV5 (Zhao et al., 2016). We only used human data.

### The Performance of Deep Belief Network–Convolutional Neural Network

We did 10-cross validation on each cancer. Area under the curve (AUC) (Cheng, 2019; Dao et al., 2020; Zhang et al., 2020) and area under the precision–recall curve (AUPR) were used to evaluate the performance of DBN–CNN. The results are shown in Table 2.

**Table 2:** The performance of deep belief network (DBN)–convolutional neural network (CNN) in 16 cancers.

**Cancer**	**Area under curve (AUC)**	**Area under precision curve (AUPR)**
Cervical cancer	0.87	0.82
Breast cancer	0.81	0.78
Colorectal cancer	0.81	0.75
Stomach cancer	0.88	0.83
Urinary bladder cancer	0.93	0.85
Lung cancer	0.79	0.82
Ovarian cancer	0.93	0.85
Thyroid cancer	0.94	0.86
Prostate cancer	0.84	0.72
Liver cancer	0.84	0.74
Pancreatic cancer	0.77	0.69
Ovarian epithelial cancer	0.90	0.87
Gallbladder cancer	0.96	0.88
Endometrial cancer	0.76	0.72
Colon cancer	0.88	0.82
Esophageal cancer	0.91	0.87

As we can see in Table 2, the performance of DBN–CNN is quite different in different cancers. This may be caused by the different sample sizes. The average AUC is 0.86 and AUPR is 0.80.

### Comparison Experiments

To verify the superior of DBN–CNN, we compared it with similar methods. Since the main function of DBN is to reduce dimension, principal component analysis (PCA) has the same function. Therefore, instead of using DBN to encode, we used PCA this time and CNN was used to classify the features after PCA. We call this method PCA–CNN. In addition, we also used the deep neural network (DNN) to replace CNN so this comparison method was called DBN–DNN.

We used these three methods to test on 16 cancers and summarized the results to get a final AUC and AUPR for each method. The receiver operating characteristic (ROC) curves are shown in Figure 4.

**Figure 5:**
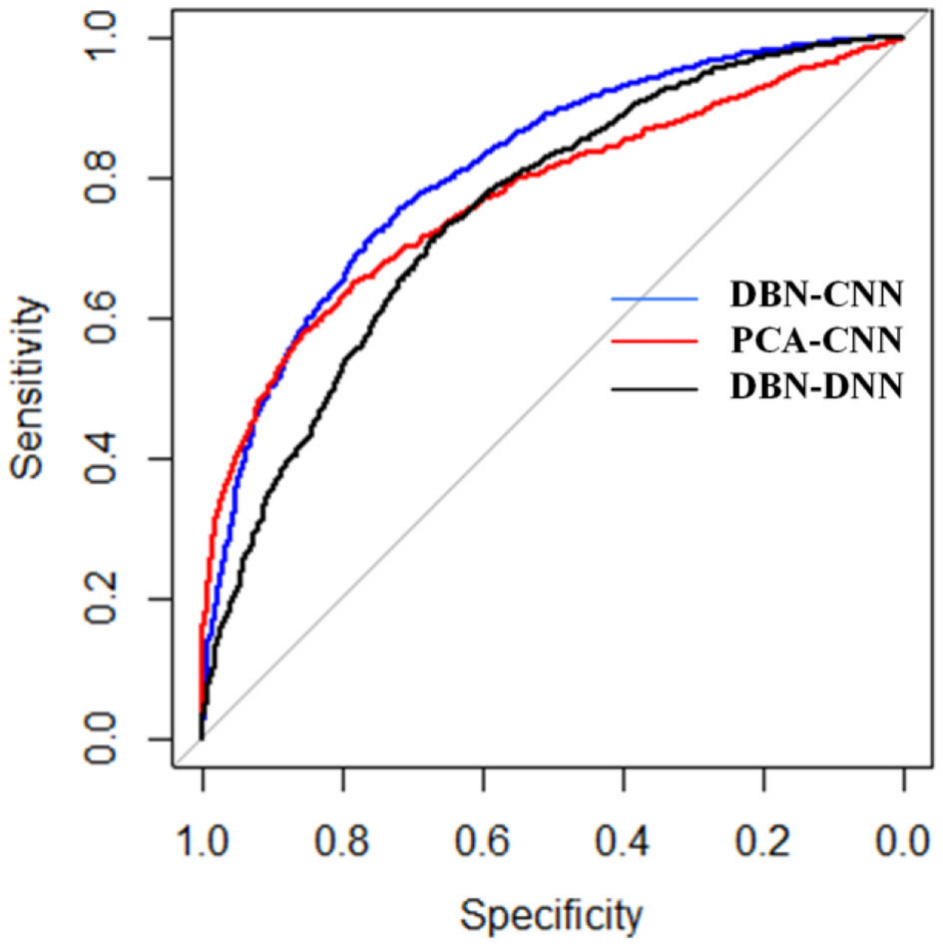
The receiver operating characteristic (ROC) curves of the three methods. DBN, deep belief network; CNN, convolutional neural network; PCA, principal component analysis.

As shown in Figure 5, the blue curve denotes the results of DBN–CNN. The red and black curves denote PCA–CNN and DBN–DNN, respectively. As we can see, DBN–CNN performed best among these three methods. The AUC of DBN–CNN is 0.81, which is better than 0.77 and 0.75 for PCA–CNN and DBN–DNN, respectively.

**Figure 6:**
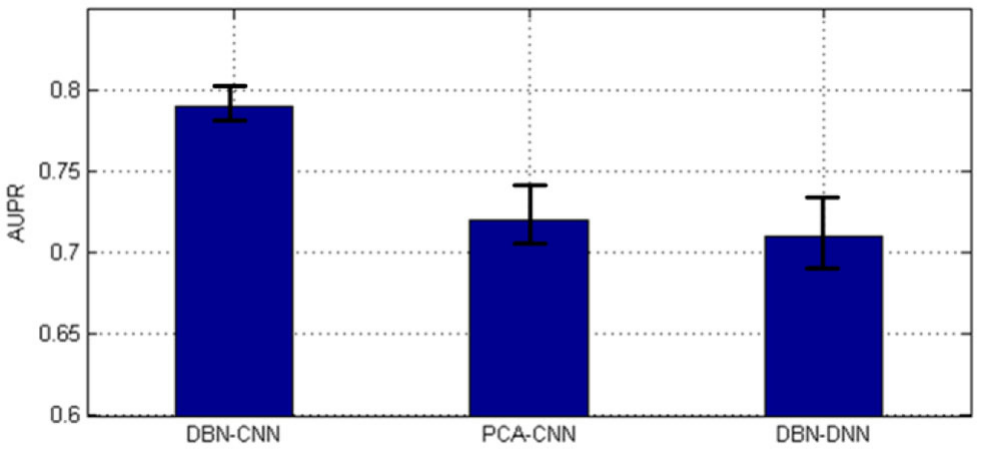
The area under the precision–recall curve (AUPR) of the three methods. DBN, deep belief network; CNN, convolutional neural network; PCA, principal component analysis.

As shown in Figure 6, the AUPR of DBN–CNN is the highest with the least standard error.

### Case Study

Liu et al. (2002) found down syndrome cell adhesion molecule - antisense RNA 1 (DSCAM-AS1) is associated with breast cancer by constructing two suppression subtracted cDNA libraries.

Martens-Uzunova et al. (2014) reported the association between H19 and bladder cancer. They also pointed out that H19 could be the biomarker of bladder cancer.

Shi et al. (2014) measured the expression level of lncRNAs-Loc554202 in breast cancer tissues and found that Loc554202 was significantly increased compared with normal control and associated with advanced pathologic stage and tumor size.

## Conclusions

Increasing evidence has shown the relationship between lncRNAs and cancers. lncRNAs could be the biomarkers to help diagnose cancer and also help researchers understand the mechanism of cancers. Compared with people's knowledge of disease-related protein coding genes, we knew few about disease-related lncRNAs. However, the biological experiments for finding disease-related lncRNAs are time-consuming and expensive.

Therefore, in this paper, we proposed a novel method for identifying cancer-related lncRNAs. We called this method “DBN–CNN,” which is a fusion of DBN and CNN. Two kinds of features were used based on the biological background. Since lncRNAs have tissue-specific expression and the expression of cancer tissues is different from normal tissues, the expression of lncRNAs in different tissues could provide important information for us to identify cancer-related lncRNAs. In addition, lncRNAs execute their regulation function by interacting with their target genes. Therefore, the target genes of lncRNAs can also be the features of lncRNAs. To encode the features, DBN was used to reduce the dimension. Finally, CNN was used to identify real cancer-related lncRNAs based on the final feature.

To verify the effectiveness of our method, we compared DBN–CNN with PCA–CNN and DBN–DNN since PCA can also reduce the dimension of features and DNN can also do classification. The results showed that DBN–CNN performed best. Finally, case studies have been done to verify the accuracy of our results. We found potential lncRNAs for 16 kinds of cancers, which can be a kind of guidance for researchers finding novel cancer-related lncRNAs.

## Data Availability Statement

The datasets presented in this study can be found in online repositories. The names of the repository/repositories and accession number(s) can be found in the article/supplementary material.

## Author Contributions

ND and GX designed the research. ZL performed the research and wrote the manuscript. YZ and XH acquired the data and reviewed and edited the manuscript. CL, XY, and JG analyzed the data. All authors reviewed the manuscript and provided comments.

## Conflict of Interest

The authors declare that the research was conducted in the absence of any commercial or financial relationships that could be construed as a potential conflict of interest.
